# Excision and Primary Anastomosis for Short Bulbar Strictures: Is It Safe to Change from the Transecting towards the Nontransecting Technique?

**DOI:** 10.1155/2018/3050537

**Published:** 2018-11-01

**Authors:** M. Waterloos, W. Verla, W. Oosterlinck, P. François, N. Lumen

**Affiliations:** ^1^Department of Urology, Ghent University Hospital, Ghent, Belgium; ^2^Department of Urology, Maria Middelares Ziekenhuis, Ghent, Belgium; ^3^Department of Urology, Centre Hospitalier de Mouscron, Mouscron, Belgium

## Abstract

**Objective:**

To explore whether it is safe to change from transecting excision and primary anastomosis (tEPA) towards nontransecting excision and primary anastomosis (ntEPA) in the treatment of short bulbar urethral strictures and to evaluate whether surgical outcomes are not negatively affected after introduction of ntEPA.

**Materials and Methods:**

Two-hundred patients with short bulbar strictures were treated by tEPA (n=112) or ntEPA (n=88) between 2001 and 2017 in a single institution. Failure rate and other surgical outcomes (complications, operation time, hospital stay, catheterization time, and extravasation at first cystography) were calculated for both groups. Potentially predictive factors for failure (including ntEPA) were analyzed using Cox regression analysis.

**Results:**

Median follow-up for the entire cohort was 76 months, 118 months, and 32 months for, respectively, tEPA and ntEPA (p<0.001). Nineteen (9.5%) patients suffered a failure, 13 (11.6%) with tEPA and 6 (6.8%) with ntEPA (p=0.333). High-grade (grade ≥3) complication rate was low (1%) and not higher with ntEPA. Median operation time, hospital stay, and catheterization time with tEPA and ntEPA were, respectively, 98 and 87 minutes, 3 and 2 days, and 14 and 9 days. None of these outcomes were negatively affected by the use of ntEPA. Diabetes and previous urethroplasty were significant predictors for failure (Hazard ratio resp. 0.165 and 0.355), whereas ntEPA was not.

**Conclusions:**

Introduction of ntEPA did not negatively affect short-term failure rate, high-grade complication rate, operation time, catheterization time, and hospital stay in the treatment of short bulbar strictures. Diabetes and previous urethroplasty are predictive factors for failure.

## 1. Introduction

The International Consultations on Urologic Diseases (ICUD) recommends urethroplasty by excision and primary anastomosis (EPA) for short and isolated bulbar urethral strictures as it provides an excellent success rate (93.8%) with a low complication rate [1]. After EPA, the diseased segment is entirely removed and replaced by own healthy urethra without the need for urethral substitution material (grafts or flaps), which is probably the reason for the high success rate. During the “classic” transecting EPA (tEPA), the corpus spongiosum containing the urethra is transected full thickness at the level of the stricture [2]. As EPA only requires excision of the narrowed urethra and the surrounding spongiofibrosis, a full thickness transection is usually not necessary. To avoid this and to preserve the dual blood supply of the urethra, Jordan et al. introduced the concept of vessel-sparing or nontransecting EPA (ntEPA) [3], later slightly modified by Andrich et al. [4]. This nontransecting variant is an attempt to reduce the surgical trauma of tEPA and several centers have introduced this technique in their reconstructive repertoire[4–6]. A prerequisite to use ntEPA is that the outcomes are at least not inferior compared to the standard technique of tEPA. Case series of ntEPA have a promising short-term success rate of 94.5-100% [3, 5–7], which is in line with the composite success rate of 93.8% for the tEPA reported by the ICUD[1]. However, indirect comparison of series is hazardous as patient and stricture characteristics, follow-up schedules, and reporting of outcomes might vary among series. Therefore, the primary objective of this study is to evaluate whether the change in practice from tEPA to ntEPA yielded surgical outcomes that are not inferior to the patient. To the best of our knowledge, this is the first paper to report this.

## 2. Material and Methods

### 2.1. Study Population

A database was collected of all male patients (n=852) who underwent urethroplasty at Ghent University Hospital starting from 2001 (start of electronic medical file). Since 2008, this collection was done prospectively. Patients who underwent EPA, either by the transecting or nontransecting technique, for isolated short bulbar strictures (ranging from the penoscrotal angle up to the urogenital diaphragm) were selected from this database until December 2017. Exclusion criteria were EPA performed for posterior or penile strictures, EPA with concomitant urethroplasty at another part of the urethra, EPA in transgender patients, and EPA in patients on clean intermittent catheterization. All patients underwent preoperative evaluation including history taking (with emphasis on stricture etiology and previous urethral interventions), clinical examination, uroflowmetry, and urethrography. According to our in-home algorithm to treat urethral strictures, EPA is the preferred technique for short (≤3cm) bulbar strictures [8]. After attendance at a masterclass on urethroplasty we became familiar with the technique and being convinced of the theoretical advantages of ntEPA, we performed our first cases in November 2011. Starting from January 2012, ntEPA became the standard technique.

### 2.2. Surgical Technique

A detailed description of the operative techniques is beyond the scope of this article as it has been published previously [6, 9]. In brief, the patient is placed in the social lithotomy position, a midline perineal incision is made, and the bulbospongiosus muscle is incised at the midline and dissected away from the corpus spongiosum containing the bulbar urethra. The bulbar urethra is circumferentially detached from the corporal bodies and mobilized from the penoscrotal angle up to the urogenital diaphragm. With tEPA, the perineal body (“centrum tendineum”) is incised for further mobilization of the ventral bulbar urethra. With this technique, the spongious tissue is transected full thickness at the level of the stricture which is marked after introduction of a metal sound through the meatus. The narrowed urethra and surrounding spongiofibrosis are fully excised, the healthy urethral ends are spatulated, and a tension-free anastomosis is made by 8 resorbable sutures 4.0. For ntEPA, the modification described by Andrich et al. was used [4]. The urethra is incised dorsally at the level of the stricture. Again, the stricture and surrounding fibrosis are excised but with preservation of the ventral spongiosum. The urethral edges are also spatulated and connected end-to-end with 8 resorbable sutures 4.0. In case of any difficulties to ensure a complete resection of the fibrosis or if the fibrosis encompasses the entire thickness of the spongious tissue, conversion to tEPA is done. The spongious tissue is closed with resorbable sutures 4.0 over the urethral anastomosis. This second layer (“spongioplasty”) is circumferential with tEPA and at the dorsolateral side with ntEPA. For both techniques, a 20Fr silicone catheter is left in place as well as a perineal suction drain.

### 2.3. Follow-Up and Evaluation

The suction drain is removed after 24-48 hours. The patient is discharged when his clinical condition allows for it, which is usually after 2 days. The catheter is removed 1 to 2 weeks later on ambulatory base if voiding cystourethrography confirms absence of extravasation [10]. In case of extravasation, the examination is repeated after one week. Follow-up including history taking and uroflowmetry was advised every 3 months during the 1st year, and annually thereafter. Surgical complications (≤90 days) were scored according to the Clavien-Dindo classification. Patients were asked to come on earlier visit if they experience obstructive urinary symptoms or had a urinary tract infection. In case of suspicion of recurrence (clinical symptoms or maximum urinary flow <15ml/s), retrograde urethrography or urethroscopy was performed. Referred patients were sent back to and followed by their local urologist. A functional definition of failure was used, namely, obstructive symptoms with the need for additional urethral instrumentation (including simple dilation) [11]. Other surgical outcomes analyzed are operation time, hospital stay, catheterization time, and extravasation at first cystography. Functional outcomes (incontinence, erectile function, and genital sensitivity) are not the scope of this study as these parameters were not systematically questioned and in those where it was done, different questionnaires were used over the years. The study was approved by the local ethics committee (EC UZG 2008/234). All operations were done by 2 surgeons (W.O., N.L.).

### 2.4. Statistical Analysis

A first analysis was done per surgical technique (tEPA versus ntEPA). As mentioned above, since 2012 ntEPA became the standard technique. However, in case of difficulties or severe spongiofibrosis, conversion to tEPA was possible. As these are presumably more complex cases, a selection bias between surgical groups since 2012 is imminent. In order to minimize this, a second analysis was done using the intention-to-treat (ITT) principle in which all conversions to tEPA since 2012 remained classified as ntEPA cases (further called “ITT-ntEPA”). Statistical tests were done using IBM™ SPSS software version 25.0. All tests were done 2-sided and a p value <0.05 indicates statistical significance. Next to descriptive statistics, categorical variables were compared using Fischer's exact test. Continuous variables were analyzed for parametric distribution using the Shapiro-Wilk test and as all variables had a nonparametric distribution, the Mann-Whitney* U* test was used for comparison. Failure-free survival (FFS) was calculated using Kaplan-Meier survival analysis with log rank statistics. To evaluate whether ntEPA was an independent predictor for failure, uni- and multivariate Cox regression analysis with calculation of the Hazard Ratio (HR) was performed. The following variables were included: type of urethroplasty (tEPA versus ntEPA), previous urethroplasty, presence of suprapubic catheter, stricture length, and diabetes. For every surgical parameter (failure rate, complication rate, operation time, hospital stay, extravasation at first cystography, and catheterization time) the null hypothesis (H_0_) was as follows: “the surgical parameter is not worse with ntEPA compared to tEPA.”

## 3. Results

### 3.1. Per Surgery Analysis

A total of 200 patients underwent EPA, 112 by tEPA and 88 by ntEPA. Median follow-up for the entire cohort was 76 months, with a significant longer follow-up for tEPA compared to ntEPA (resp., 118 versus 32 months; p<0.001). There were no significant differences between both groups for patient's age, presence of diabetes, stricture etiology, presence of a suprapubic catheter, and previous urethroplasty (Table 1). Median stricture length with tEPA and ntEPA was 1,5 and 1,25 cm, respectively (p=0.004). The following hypotheses concerning surgical outcomes were evaluated (Table 2).

#### 3.1.1. *H*_0_: Operation Time Is Not Longer with ntEPA Compared to tEPA

Median operation time with tEPA and ntEPA was, respectively, 98 and 87 minutes (p<0.001). The null hypothesis cannot be rejected.

#### 3.1.2. *H*_0_: Hospital Stay Is Not Longer with ntEPA Compared to tEPA

Patients treated by tEPA had a median hospital stay of 3 days compared to 2 days with ntEPA (p<0.001). The null hypothesis cannot be rejected.

#### 3.1.3. *H*_0_: ntEPA Is Not Associated with More Extravasation at First Cystography Compared to tEPA and There Is No Longer Catheterization Time with ntEPA Compared to tEPA

Extravasation at first cystography requiring catheter reinsertion was observed in 5.4% and 6.8% of patients treated, respectively, by tEPA and ntEPA (p=0.768). Median catheterization time with tEPA and ntEPA was, respectively, 14 and 9 days (p<0.001). The null hypothesis cannot be rejected.

#### 3.1.4. *H*_0_: ntEPA Is Not Associated with More Complications Compared to tEPA

No complications were reported in 89.3 and 79.5% for, respectively, tEPA and ntEPA (p=0.147). Low-grade complication rate was not significantly different among groups. Two patients (1%) suffered a grade 3 complication (requiring intervention), one in each group. The null hypothesis cannot be rejected.

#### 3.1.5. *H*_0_: ntEPA Has No Higher Failure Rate Compared to tEPA

13 (11.6%) and 6 (6.8%) patients, respectively, suffered a failure with tEPA and ntEPA (p=0.333). The estimated 1- and 3-year FFS is 98.2 and 95.5% for tEPA and 95.5 and 95.5% for ntEPA (p=0.356) (Table 2). The null hypothesis cannot be rejected.

### 3.2. Intention-to-Treat (ITT) Analysis

Patient and stricture characteristics did not significantly differ between these 2 cohorts (Table 3). As mentioned above, all patients in the ITT-tEPA cohort (n=101) underwent tEPA. However, conversion towards tEPA was performed in 11 of 99 (11.1%) patients of the ITT-ntEPA cohort.  Table 4 provides information about the characteristics of the patients converted to tEPA and those treated by ntEPA. In the ITT-ntEPA cohort, patients finally treated with tEPA had a median stricture length of 2 cm compared to 1,25 cm for ntEPA (p=0.019) whereas other preoperative characteristics were comparable. Median operation time for ITT-tEPA and ITT-ntEPA was, respectively, 95 and 88 minutes (p<0.009). in the ITT-ntEPA cohort, patients finally treated by tEPA had a median operation time of 155 minutes compared to 87 minutes with ntEPA (p=0.01). Median hospital stay was 3 and 2 days for, respectively, ITT-tEPA and ITT-ntEPA (p<0.001). In the ITT-ntEPA cohort, patients finally treated by tEPA and ntEPA both had a median hospital stay of 2 days (p=0.088). Extravasation at first cystography was reported in 4% and 8.1% of ITT-tEPA and ITT-ntEPA cases, respectively (p=0.248). Median catheterization time with ITT-tEPA and ITT-ntEPA was, respectively, 14 and 9 days (p<0.001). In the ITT-ntEPA cohort, patients treated by tEPA had a longer catheterization time compared to ntEPA (15 versus 9 days; p=0.005). More low-grade (G1-G2) complications were reported in the ITT-ntEPA cohort compared to ITT-tEPA (20,2% versus 7,9%; p=0.024). In the ITT-ntEPA cohort, patients finally treated by tEPA had a complication rate of 27,3% compared to 20,5% for patients treated by ntEPA (p=0.339). 12 (11.9%) and 7 (7.1%) patients, respectively, suffered a failure with ITT-tEPA and ITT-ntEPA (p=0.336). The estimated 1- and 3-year FFS are 98 and 95% for tEPA and 96 and 96% for ntEPA (p=0.256).

### 3.3. Additional Analyses

In total, 19 (9.5%) patients suffered a failure. Of the 19 failed cases, 6 (31.6%) cases were observed within the 1st year, 6 (31.6%) between the 2nd and 5th year and 7 (36.8%) after the 5th postoperative year. For tEPA, 2 (15.4%), 4 (30,8%), and 7 (53,8%) failures were detected within the 1st year, between the 2nd and 5th year, and after 5 years, respectively. For ntEAP, 4 (66.7%) cases were detected during the 1st year after operation and 2 (33.3%) between the 2nd and 5th year.

Cox regression analysis could not identify ntEPA as a predictor for failure (Table 5). In univariate analysis, previous urethroplasty (HR 0.369; p=0.044) and diabetes (HR 0.185; p=0.009) were predictive for failure. Both previous urethroplasty (HR 0.355; p=0.043) and diabetes (HR 0.165; p=0.006) remained negative predictive factors in multivariate analysis.

## 4. Discussion

The success rate of 88.4% for tEPA in this series might appear somewhat lower compared to the 93.8% composite success rate for tEPA reported in the ICUD-review [1]. However, the median follow-up of 115 months in this paper is substantially longer compared to the papers included in that review [1]. Andrich et al. reported an 87% success rate after 10-year follow-up [12]. Although this series reported durable results on the long term with most of the recurrences occurring with the first years after surgery [12], this could not be confirmed by the present series as 53.8% of failures with tEPA were found even after the 5th postoperative year. In two other series, where time-related events are available, a steady decline in the success rate of tEPA was observed as well [13, 14]. As for substitution urethroplasty, this indicates that EPA also needs prolonged follow-up as late recurrences are possible. Some of our late failures were detected on occasion in an asymptomatic patient for which access to the bladder was needed (e.g., urethral catheter during surgery and cystoscopy because of hematuria). It has indeed been described that a stricture only becomes symptomatic once the urethral diameter is less than 10Fr. It is likely that a strict follow-up schedule with standard cystoscopy would have detected these failures earlier [11]. Some of the late failures might also be attributed to progression of the stricture disease as almost 20% of patients already underwent previous urethroplasty. The shorter follow-up with ntEPA in this series is explained by the change in practice since 2012 where it became the standard technique. The 93.2% success rate with ntEPA is in line with previous reports [3–7]. Estimated 1- and 3-FFS could not demonstrate inferiority of ntEPA versus tEPA nor could uni- and multivariate Cox regression analysis identify ntEPA as an independent predictive factor for failure. With ntEPA, 2 failures were detected between the 2nd and 5th postoperative year, also underlining the need for prolonged follow-up to evaluate whether this noninferiority remains on the long term (>5 years follow-up). With ntEPA, the operation time was on average 11 minutes shorter. With ntEPA, no need for ventral dissection deeper than the perineal body is needed which saves time. Furthermore, full transection of the corpus spongiosum with tEPA leads to substantial bleeding through the bulbar arteries with need for additional hemostasis (and time to achieve this). On the other hand, we perceive that the anastomosis itself is somewhat more difficult to perform and more time-consuming with ntEPA. However, other factors might bias operation time. By the standard introduction of ntEPA in 2012, both surgeons already had a large experience with urethral anatomy and urethroplasty. This experience probably has facilitated the introduction of ntEPA. In the earlier stages when uniformly tEPA was performed, this experience was less and surgery could have taken more time. Furthermore, since 2012 an important selection bias is present at the expense of tEPA: the more complex cases are still treated with tEPA and this complexity might account for a longer operation time. Nevertheless, even with ITT-analysis, operation time remained in favor of ntEPA. At least, this indicates that a shift in practice towards the use of ntEPA does not negatively affect operation time in surgeons already proficient with tEPA. The more complex nature of tEPA cases since 2012 might also be apparent by the longer stricture length and the longer catheterization time compared to the contemporary ntEPA cases. This selection bias might be the reason why strictures treated by ntEPA were shorter in the per surgery analysis but no longer in the ITT-analysis. This selection bias might in part explain the longer catheterization time with tEPA. However, the longer catheterization time is undoubtedly related to a change in our practice for catheter stay since 2010 when it was decided to remove the catheter after 1 week for simple cases (whereas this was 2 weeks before) [10]. With ntEPA, a one-day shorter hospital stay was observed. Although this might indicate a quicker recovery with ntEPA, this cannot by assumed as such. In recent years, budgetary reasons have urged us to discharge the patients as early as possible which probably attributed towards the shorter hospital stay since 2012. The observation that the tEPA cases since 2012 had an equal hospital stay despite a probably more complex stricture supports the latter hypothesis. The complication rate in this series is low and confirms the finding of other colleagues [5, 14]. High-grade (≥grade 3) complications were not more frequent with ntEPA. With ITT-analysis (but not per surgery analysis), low-grade complications were somewhat more frequent with ntEPA. This is likely due to the mainly retrospective data collection with risk of underreporting of low-grade complications in tEPA versus the prospective data collection with ntEPA. Nevertheless, this observation must raise a concern and warrants further evaluation.

Despite introduction of ntEPA, we needed to convert towards a tEPA in approximately 10% of cases. A more distal location of the stricture within the bulbar urethra was not a reason for conversion to tEPA in this series. The main reason for conversion was extensive spongiofibrosis (“full thickness”) in which it was no longer valuable to spare the ventral spongious tissue. This conversion to tEPA is not at all jeopardized by an approach to go for ntEPA as all initial surgical steps are the same. From this series, it is clear that tEPA must remain in the repertoire of the urethral surgeon. Furthermore, tEPA remains indispensable in the delayed treatment of pelvic fracture related injuries [15, 16] and iatrogenic posterior urethral injuries [17]. However, the applicability of ntEPA for posterior strictures is currently explored as well [6, 18].

The aim of ntEPA is to reduce the surgical trauma with preservation of the dual blood supply of the urethra. This might offer an advantage for subsequent urethral interventions, e.g., redo-urethroplasty with free graft in which a well-vascularized graft bed is essential or implantation of an artificial urinary sphincter with less risk of cuff erosion [3]. Furthermore, ntEPA might offer a benefit regarding the reported vascular deficiency of the glans penis and the risk of erectile dysfunction with tEPA [19, 20]. The present dataset lacks information to evaluate these potential advantages. Nevertheless, despite the theoretical benefit, at least a transient decline in erectile function in 6-21.9% of cases has already been reported with ntEPA as well [4–7].

Diabetes and previous urethroplasty were identified as independent predictors for failure. With both techniques of EPA and the associated need for extensive mobilization of the bulbar urethra, the “3th” vascular supply (small arterial connections between the corporal bodies and the corpus spongiosum) of the urethra is sacrificed. Diabetes with its associated microangiopathy further increases the risk of ischemia at the urethral ends which is a reason for failure of the anastomosis [21]. In addition, diabetes might be a contributing factor in the development of ischemic strictures, which might explain some late failures. Diabetes as risk factor for failure was identified by another series as well [21]. A previous failed urethroplasty usually reflects a more complex urethral pathology with a higher risk of failure [22]. EPA for a failure after previous urethroplasty is possible in case of a previous EPA in which the urethral mobilization was insufficient for tension-free anastomosis (technical error). EPA is also possible for a short recurrence after graft urethroplasty, usually at one of the ends of the graft [23]. Other series have also identified previous urethroplasty as a negative predictive factor [14, 21], whilst others have not [23].

This study has several limitations. Until 2008, data collection was retrospective with its inherent risk of bias. Although a follow-up schedule is proposed to the patients and the referring urologists, this is not systematically followed. This might also explain delayed detection of failure or underreporting of (minor) complications. A functional definition of failure was used, but at the moment, an anatomical definition is advised as it is more accurate and objective [11]. Validated patient reported outcome measures as suggested by Jackson et al. [24] were not systematically used, as it lasted to 2017 until a Dutch validation was available [25]. This prohibits any meaningful further evaluation. The follow-up of ntEPA is relatively short. Another important limitation is that this paper is an evaluation of basically 2 noncontemporary cohorts. Changes in practice, increasing surgical experience, selection of more challenging cases, etc. might have a major impact on outcome parameters. Therefore, any direct comparison of these 2 cohorts must be avoided. To overcome all the above-mentioned shortcomings, it is necessary to conduct a prospective randomized study comparing tEPA with ntEPA evaluating surgical and functional outcomes using a strict protocol. Because important surgical parameters were not negatively affected in this series after introduction of ntEPA, it appears justified to start up such a trial. In this perspective and to elucidate the definitive role of ntEPA, our center has initiated the VeSpAR-trial: a prospective randomized controlled trial comparing  Vessel-Sparing  Anastomotic  Repair and transecting anastomotic repair in isolated short bulbar urethral strictures (ClinicalTrials.gov NCT03572348).

## 5. Conclusion

Introduction of ntEPA for short bulbar strictures by experienced urethral surgeons does not negatively affect short-term failure rate, high-grade complication rate, operation time, hospital stay, and catheterization time. Late recurrences are possible with both types of EPA underlining the need for continued follow-up in these patients. tEPA must remain in the surgical repertoire for challenging cases. Diabetes and previous urethroplasty are independent predictors for failure of EPA.

## Figures and Tables

**Table 1:** Patient and stricture characteristics (IQR: interquartile range; tEPA: transecting excision and primary anastomosis; ntEPA: nontransecting excision and primary anastomosis).

	Total (n=200)	tEPA (n=112)	ntEPA (n=88)	p-value
age (years); median (IQR)	49 (32-65)	49 (34-66)	47 (30-64)	0,216
follow-up (months); median (IQR)	76 (32-122)	118 (93-148)	32 (17-57)	**<0,001**
stricture length (cm); median (IQR)	1,5 (1-2)	1,5 (1-2)	1,25 (1-2)	**0,004**
diabetes; n(%)	11 (5,5%)	6 (5,4%)	5 (5,7%)	1
presence of suprapubic catheter; n(%)	44 (22%)	29 (25,9%)	15 (17%)	0,169
previous urethroplasty; n(%)	37 (18,5%)	19 (17%)	18 (20,5%)	0,584
etiology; n(%)				
*idiopathic/congenital*	102 (51%)	52 (46,4%)	50 (56,8%)	0,508
*iatrogenic*	72 (36%)	43 (38,4%)	29 (33%)	
*external trauma*	20 (10%)	13 (11,6%)	7 (8%)	
*inflammatory*	6 (3%)	4 (3,6%)	2 (2,3%)	

**Table 2:** Surgical outcome per surgery technique (IQR: interquartile range; FFS: failure-free survival; tEPA: transecting excision and primary anastomosis; ntEAP: nontransecting excision and primary anastomosis).

	Total (n=200)	tEPA (n=112)	ntEPA (n=88)	p-value
operation time (mintues); median (IQR)	92 (79-108)	98 (80-115)	87 (71-100)	**<0,001**
hospital stay (days); median (IQR)	2 (2-3)	3 (2-4)	2 (1-2)	**<0,001**
extravasation at first cystography; n(%)	12 (6%)	6 (5,4%)	6 (6,8%)	0,768
catheterization time (days); median (IQR)	13 (9-14)	14 (13-15)	9 (8-13)	**<0,001**
complications; n(%)				
none	170 (85%)	100 (89,3%)	70 (79,5%)	0,147
G1	20 (10%)	8 (7,1%)	12 (13,6%)	
G2	8 (4%)	3 (2,7%)	5 (5,7%)	
G3	2 (1%)	1 (0,9%)	1 (1,1%)	
Failure, n(%)	19 (9,5%)	13 (11,6%)	6 (6,8%)	0,333
Estimated failure free survival, % (standard deviation)				
1y-FFS	97 (±1,2)%	98,2 (±1,3)%	95,5 (±2,2)%	0,356
3y-FFS	95,2 (±1,6)%	95,5 (±2)%	95,5 (±2,2)%	
10y-FFS	85,6 (±3,5)%	86,9 (±3,7)%	NR	

**Table 3:** Surgical outcome per intention-to-treat cohort (IQR: interquartile range; FFS: failure-free survival; ITT-tEPA: intention-to-treat transecting excision and primary anastomosis; ITT-ntEAP: intention-to-treat nontransecting excision and primary anastomosis; NA: not available).

	ITT-tEPA (n=101)	ITT-ntEPA (n=99)	p-value
follow-up (months); median (IQR)	122 (97-150)	33 (17-59)	**<0,001**
age (years); median (IQR)	50 (34-67)	44 (31-63)	0,102
stricture length (cm); median (IQR)	1,5 (1-2)	1,5 (1-2)	0,07
diabetes; n(%)	6 (5,9%)	5 (5,1%)	1
presence of suprapubic catheter; n(%)	26 (25,7%)	18 (18,2%)	0,233
operation time (minutes); median (IQR)	95 (80-110)	88 (73-100)	**0,009**
previous urethroplasty; n(%)	15 (14,9%)	22 (22,2%)	0,205
hospital stay (days); median (IQR)	3 (2-4)	2 (1-2)	**<0,001**
extravasation at first cystography; n(%)	4 (4%)	8 (8,1%)	0,248
catheterisation time (days); n(%)	14 (13-14)	9 (8-14)	**<0,001**
failure; n(%)	12 (11,9%)	7 (7,1%)	0,336
complications; n(%)			
none	92 (91,1%)	78 (78,8%)	**0,024**
G1	7 (6,9%)	13 (13,1%)	
G2	1 (1%)	7 (7,1%)	
G3	1 (1%)	1 (1%)	
Estimated failure free survival, % (standard deviation)			
1y-FFS	98 (±1,4)%	96 (±2)%	0,256
3y-FFS	95 (±2,2)%	96 (±2)%	
10y-FFS	87,4 (±3,7)%	NA	

**Table 4:** Characteristics and surgical outcomes of patients treated by tEPA and ntEPA in the intention-to-treat ntEPA cohort (IQR: interquartile range; FFS: failure-free survival; ITT-tEPA: intention-to-treat transecting excision and primary anastomosis; ITT-ntEAP: intention-to-treat nontransecting excision and primary anastomosis; NA: not available).

	tEPA (n=11)	ntEPA (n=88)	p-value
follow-up (months); median (IQR)	36 (23-73)	32 (17-57)	0,308
age (years); median (IQR)	44 (36-52)	47 (30-64)	0,676
stricture length (cm); median (IQR)	2 (1,25-2,5)	1,25 (1-2)	**0,019**
diabetes; n(%)	0 (0%)	5 (5,7%)	1
presence of suprapubic catheter; n(%)	3 (27,3%)	15 (17%)	0,415
previous urethroplasty; n(%)	4 (36,5%)	18 (20,5%)	0,256
operation time (minutes); median (IQR)	115 (88-158)	87 (71-100)	**0,01**
hospital stay (days); median (IQR)	2 (2-3)	2 (1-2)	0,088
extravasation at first cystography; n(%)	2 (18,2%)	6 (6,8%)	0,217
catheterization time (days); median (IQR)	15 (12-15)	9 (8-13)	**0,005**
failure; n(%)	1 (9,1%)	6 (6,8%)	0,574
complications; n(%)			
none	8 (72,7%)	70 (79,5%)	0,339
G1	1 (9,1%)	12 (13,6%)	
G2	2 (18,2%)	5 (5,7%)	
G3	0 (0%)	1 (1,1%)	

**Table 5:** Uni-and multivariate Cox regression analysis to predict for failure (HR: Hazard Ratio; CI: confidentiality interval; tEPA: transecting excision and primary anastomosis; ntEPA: nontransecting excision and primary anastomosis).

	Univariate			Multivariate		
	HR	95% CI	p-value	HR	95% CI	p-value
type of urethroplasty (tEPA vs ntEPA)	0,593	0,193-1,822	0,361	0,671	0,212-2,122	0,497
previous urethroplasty (no versus yes)	0,368	0,139-0,973	**0,044**	0,355	0,130-0,970	**0,043**
suprapubic catheter (no versus yes)	1,613	0,469-5,539	0,448	1,468	0,409-5,259	0,556
Stricture length	0,784	0,383-1,605	0,505	0,743	0,340-1,623	0,456
diabetes (no versus yes)	0,185	0,053-0,651	**0,009**	0,165	0,046-0,596	**0,006**

## Data Availability

The data used to support the findings of this study are available from the corresponding author upon request.
